# Biomechanical evaluation of predictive parameters of progression in adolescent isthmic spondylolisthesis: a computer modeling and simulation study

**DOI:** 10.1186/1748-7161-7-2

**Published:** 2012-01-18

**Authors:** Amandine Sevrain, Carl-Eric Aubin, Hicham Gharbi, Xiaoyu Wang, Hubert Labelle

**Affiliations:** 1 École Polytechnique, Universite de Montreal, P.O. Box 6079, Station Centre-Ville, Montréal (Québec), H3C 3A7 CANADA; 2 Research Center, Sainte-Justine University Hospital Center of Universite de Montreal, 3175, Cote Sainte-Catherine Rd, Montréal (Québec), H3T 1C5 CANADA

**Keywords:** Isthmic spondylolisthesis, finite element model, pelvic incidence, biomechanical model

## Abstract

**Background:**

Pelvic incidence, sacral slope and slip percentage have been shown to be important predicting factors for assessing the risk of progression of low- and high-grade spondylolisthesis. Biomechanical factors, which affect the stress distribution and the mechanisms involved in the vertebral slippage, may also influence the risk of progression, but they are still not well known. The objective was to biomechanically evaluate how geometric sacral parameters influence shear and normal stress at the lumbosacral junction in spondylolisthesis.

**Methods:**

A finite element model of a low-grade L5-S1 spondylolisthesis was constructed, including the morphology of the spine, pelvis and rib cage based on measurements from biplanar radiographs of a patient. Variations provided on this model aimed to study the effects on low grade spondylolisthesis as well as reproduce high grade spondylolisthesis. Normal and shear stresses at the lumbosacral junction were analyzed under various pelvic incidences, sacral slopes and slip percentages. Their influence on progression risk was statistically analyzed using a one-way analysis of variance.

**Results:**

Stresses were mainly concentrated on the growth plate of S1, on the intervertebral disc of L5-S1, and ahead the sacral dome for low grade spondylolisthesis. For high grade spondylolisthesis, more important compression and shear stresses were seen in the anterior part of the growth plate and disc as compared to the lateral and posterior areas. Stress magnitudes over this area increased with slip percentage, sacral slope and pelvic incidence. Strong correlations were found between pelvic incidence and the resulting compression and shear stresses in the growth plate and intervertebral disc at the L5-S1 junction.

**Conclusions:**

Progression of the slippage is mostly affected by a movement and an increase of stresses at the lumbosacral junction in accordance with spino-pelvic parameters. The statistical results provide evidence that pelvic incidence is a predictive parameter to determine progression in isthmic spondylolisthesis.

## Background

Spondylolisthesis is a spinal condition characterized by a posteroanterior slippage of one vertebra over the vertebra immediately below [1-4]. The biomechanics of its occurrence and progression is yet to be fully studied. The knowledge on its biomechanics is essential for its clinical prediction and the improvement of its treatment. Spondylolisthesis was thought to be closely related to spondylolysis which is a unilateral or bilateral pars defect of a vertebra that affects 5-6% of the population [5]. Other factors, such as disc herniation or changes in spinopelvic morphology, also have an important role in the occurrence and development of spondylolisthesis [1-4]. Approximately 80% of patients with spondylolysis at L5 have the isthmic type of spondylolisthesis, and 20% of these same patients show a slippage that exceeds 25% [6]. In pediatric patients, elevated stress in the structures surrounding the growth plate may cause epiphyseal separation, apophyseal bony ring fracture, slippage at the growth plate without provoking disc degeneration and formation of a sacral dome, but the etiology and pathomechanisms of spondylolisthesis remain unclear [7-12].

**Figure 1:**
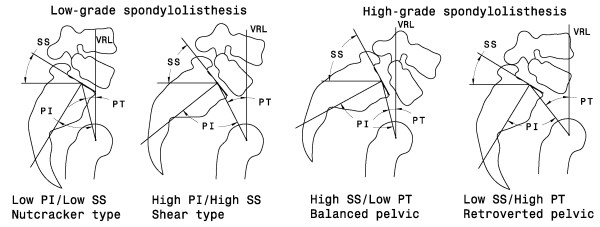
**Classification of low-grade and high-grade spondylolisthesis**.

Studies have been reported on how the occurrence and development of spondylolisthesis might be influenced by various spinal parameters. Retrospective and prospective investigations have been performed to determine spinal parameters that may increase the risk of spondylolisthesis progression using radiographic measurements. Pelvic incidence (PI) has been shown to be an important predicting factor for assessing the risk of progression of low-grade spondylolisthesis [1,2]. In high-grade spondylolisthesis, sagittal plane pelvic orientation parameters, such as sacral slope (SS) and pelvic tilt (PT), are more relevant since the PI is always high [13]. Spinal and lumbosacral parameters, essential to maintain global sagittal balance (such as slip angle, the lumbar index or the sacral contour), are thought to be secondary changes as their role is related to the degree of dysplasia rather than the cause of slippage [1,14,15]. Mac-Thiong et al. proposed a clinically oriented classification method, which detailed that in low-grade spondylolisthesis, patients with high PI/high SS were classified as "shear-type", and those with low PI/low SS were classified as "nutcracker-type" [7]. In high grade spondylolisthesis, patients with high SS/low PT were classified as "balanced pelvis", while those with low SS/high PT were classified as "retroverted pelvis" (Figure 1). Since spondylolisthesis is mainly assessed using radiographic measurements, forces responsible for the progression of the deformity remain unclear.

Discussions were also reported on the biomechanical aspects of the occurrence and progression of the spondylolisthesis. Mac-Thiong suggested that dysplastic changes affect the direction and magnitude of stress and so increase the risk of progression. Therefore, the presence of different patterns of sagittal spinopelvic balance suggests that biomechanical factors may influence the risk of progression in spondylolisthesis. Furthermore, a mechanism of slippage through the growth plate has been documented after a physis stress fracture of the vertebral body, while others found that the slippage occurs at the disc level [8,9,11,16-18].

Several finite element models of spondylolysis and spondylolisthesis have been reported in the literature which were aimed at understanding of the biomechanics of spondylolisthesis [3,8,9,16,19-22]. However most of these studies did not take into account the overall sagittal balance, nor the influence of the surrounding musculature, which affects the stress distribution of the anatomical structures under consideration, and the mechanisms involved in the slippage for the low-grade spondylolisthesis. Therefore, several biomechanical factors and important information are still missing in the current finite element models; the mechanism of progression from low grade to high grade spondylolisthesis in adolescents remains poorly understood since no longitudinal biomechanical studies have been performed.

The objective of this study was to assess the mechanisms involved at the lumbosacral junction in the progression of slippage from low to high grade spondylolisthesis and identify the spino-pelvic parameters that are predictive of the progression.

## Methods

**Figure 2:**
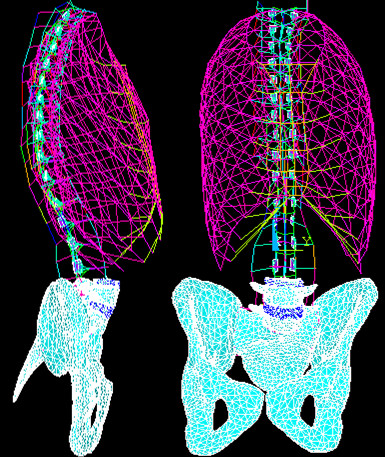
**Finite Element Model**.

The geometry of the finite element model (FEM) was constructed on the basis of patient specific characteristics obtained from a multi-view radiographic reconstruction technique. This method provided 3D coordinates of 17 points per vertebra, 11 per rib and 23 on the pelvis which were then computed using a self-calibration and optimization algorithm [23,24]. A detailed geometric model of normal vertebrae was then transformed and adjusted to match those landmarks using a dual kriging free form deformation technique. The accuracy of this reconstruction technique is 3.3 mm on average [24]. The progression mechanism was not studied from the normal state, but from a low-grade to high-grade spondylolisthesis. The patient under consideration (age = 14 years old, height = 157.2 cm, weight = 45.5 kg) possesses a low-grade spondylolisthesis (Grade II) with a PI = 61° and a SS = 52°. The resulting FEM consisted of approximately 93,000 elements governed by linear elastic behaviour (Figure 2). The model was composed of a simplified FEM with beam-type elements for the segment T1-L3 and the rib cage and a detailed volumetric model for the L4-pelvis segment [22,25-27]. The simplified FEM includes 1050 beam elements to represent the vertebrae, the intervertebral discs, the ribs, the costal cartilages and the sternum. Shells, springs and non linear contact elements were used to represent the ligaments. The detailed model includes the following: cortical shell, trabecular bone, bilateral lysis in the pars of L5, sacral dome conformed to the patient's physiology, annulus fibrosus, nucleus pulposus, all spinal ligaments, and vertebral growth plates. More specifically, the vertebral growth plates were constructed in three sections in accordance with physiological findings (a sensitive zone, a newly formed bone layer and a transition zone) [28,29]. The material properties of these anatomical partitions reflect findings from published studies (Additional file 1).

**Figure 3:**
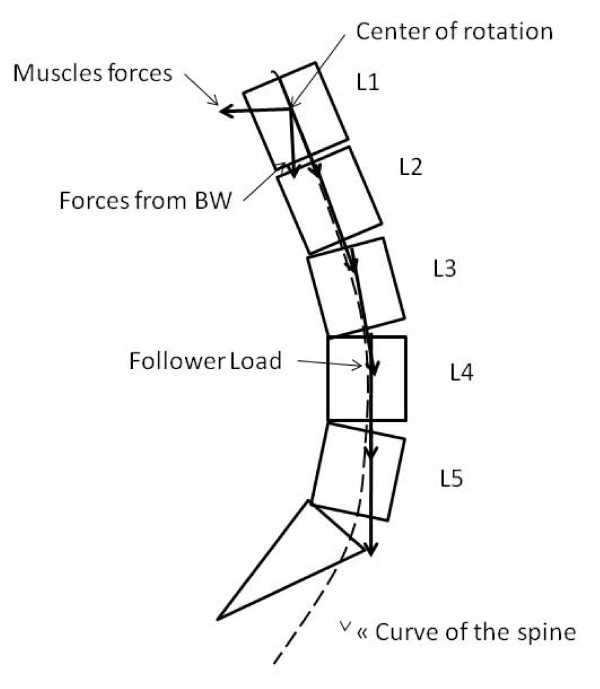
**Schematic of the follower load showing the load path "following" the lordotic curvature of the lumbar spine**.

The applied spinal forces are based on load distributions, as reported by Schultz et al., and defined by a body weight (BW) distribution on each vertebral bodies (Additional file 2) [30]. A "follower load" based on the mathematical model of Patwardhanwas was adopted in this model [31,32]. The vector sum of muscle and gravity forces produced a single internal force vector that acted tangent to the curvature of the spine and acted through each segmental centroid, "following" the kyphotic and lordotic curvature of the spine (Figure 3). The boundary conditions of the model were provided by a torsion spring at the acetabulum while T1 was blocked transversally to represent a standing posture [33].

Three spino-pelvic parameters were parameterized in the model for the aim of the study. The slip percentage was defined by an offset of the pelvis nodes in the local coordinate system of the inferior plate of L5. The pelvic incidence and sacral slopes were defined using a rotation matrix which revolved around the center of the S1 plate, with respect to the sacrum and pelvis nodes.

In order to evaluate the influence and interactions of the PI, SS and slip percentage in the progression of low-grade to high-grade spondylolisthesis, a 23 factorial design was used. The conditions of the eight FEM simulations required by this design are defined in the additional file 3. However, two of these experiments were not representative of a spondylolisthesis. As a result, a new experimental design with constraint equations was used to eliminate these cases resulting in ten plausible low and high grade spondylolisthesis configurations to simulate.

Normal and shear stress in the sagittal plane were examined, with a detailed analysis of the stress at the anterior zone of the intervertebral disc L5-S1 and of the vertebral growth plate of S1. All stresses were quantified in the local referential system described by the middle of the growth plate of L5 as origin, the xy-plan is defined on the growth plate, y-axis oriented in the sagittal plane and the z-axis is normal to the plan in direction opposed to the gravity. A negative value of the normal stress signifies that it is in compression, whereas a positive value represents tension. The normal stress is associated to the growth modulation of the growth plate. A positive value of the shear stress indicates an increase of the risk of slippage, while a negative value decreases this risk.

Statistical analyses were performed using StatSoft's STATISTICA^® ^software to determine if correlations exist between spino-pelvic parameters and stresses in the growth plate in conjunction with those measured in the intervertebral disc of the lumbosacral region. The correlations of greatest interest were investigated using an ANOVA test (with a level of significance set at p = 0.05) in order to explore existing relations between the spino-pelvic parameters and stresses in the spondylolisthesis progression.

Prior to the analyses, the model was validated using the published data of Sairyo et al. [8]. The model was adapted to compare the maximal normal stress of the growth plate and the endplate of L5 with those measured by Sairyo et al. [8,9]. To do so, a low grade spondylolisthesis shear type (PI = 61°, SS = 52°, PT = 9°) was reconstructed without sacral dome. The normal stresses in the growth plate and endplate were evaluated under physiological loading conditions using a follower load technique (Figure 2).

## Results

For the validation study, the maximal normal stress in the sagittal plane was 1.5 MPa for the growth plate of L5 and 5.34 MPa for the endplate. The difference with the results of Sairyo was 1.9 MPa and 5.8 MPa respectively for the maximal normal stress for the growth plate and for the endplate [9].

**Figure 4:**
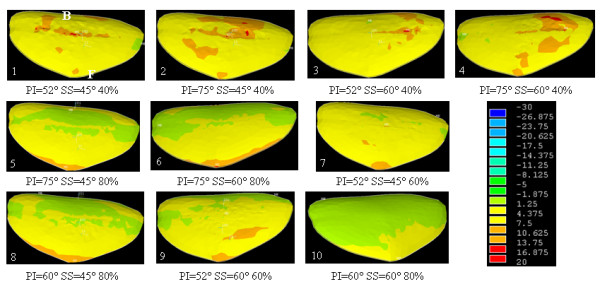
**Shear stress (MPa) on the transverse view of the growth plate of S1 for all cases of the design of experiments (B and F represent respectively the posterior and the anterior regions)**.

**Figure 5:**
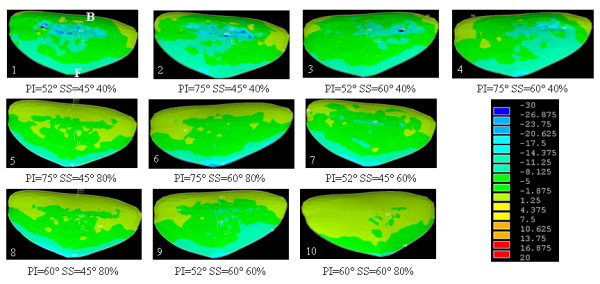
**Compression stress (MPa) on the transverse view of the growth plate of S1 for all cases of the design of experiments (B and F represent respectively the posterior and the anterior regions)**.

**Figure 6:**
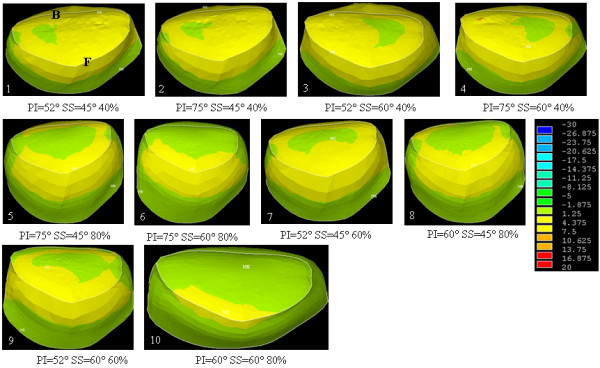
**Compression stress (MPa) on the transverse view of the intervertebral disc of L5-S1 for all cases of the design of experiments (B and F represent respectively the posterior and the anterior regions)**.

The study of low grade spondylolisthesis revealed significant differences in terms of stress distribution within the growth plate and the intervertebral disc in a spine defined by a lumbosacral junction between the nutcracker- (PI = 52°, SS = 45°) and shear-type (PI = 75°, SS = 60°) cases (Additional file 4: cases 1 and 4). Within the growth plate of the shear-type model, the shear and compression stresses were 26% and 16% higher than that of the nutcracker-type respectively. In the intervertebral disc, the shear stress measured was 5.6% higher than that of the nutcracker-type. Therefore, there was an average 16% increase of stresses between the nutcracker- and shear-type models. The elevated stresses in low grade cases were mostly located around the dome-shaped area of the growth plate and intervertebral disc (Figures 4, 5 and 6). More precisely, there was an important concentration of shear and compression stresses at the center of the growth plate, especially in cases 1 and 2 (Figures 4 and 5). The stress concentration was shifted to the right side of the growth plate in cases 3 and 4 (Figures 4 and 5). However, the variation of the stress distribution in the intervertebral disc was homogeneous in every low grade case except the top of the dome shaped area which returned a lower stress magnitude (Figure 6, cases 1 to 4).

With the simulated high-grade configuration (slip percentage of 80%), elevated stresses were located at the anterior part of the growth plate and intervertebral disc (Figures 4, 5 and 6: cases 5, 6, 8 and 10) in comparison with the low grade configurations. There was important shear stress also in the posterior part of the disc for two cases (SS = 45° and PI = 75° or PI = 60°) (Figure 6: cases 5 and 8). In the additional file 5 there were two cases (PI = 60° and SS = 45° or 60°) of high grade spondylolisthesis which had a distinct shear stress in the intervertebral disc (cases 8 and 10) while the other cases were homogenous.

Within the high-grade configurations (slip percentage of 60%), the stress difference was important between two cases (Additional file 4: cases 7 and 9) that have a different SS. In the growth plate, the shear and compression stresses were respectively 12% and 14% higher for the case with a SS at 45° than the one at 60°. The shear and compression stresses were more located on the anterior part of the growth plate for the case with the smaller sacral slope (Figures 4 and 5: cases 7 and 9). The difference was less important for the shear stress in the intervertebral disc, as there was just a difference of 0.1 MPa in the two values (Additional file 4: cases 7 and 9). However, for case 7 with SS = 45°, the compression distribution is the same of a low grade with more compression stress on the sacral dome (Figure 6: cases 7 and 9).

The pelvis balance in high grade configurations has a significant impact on the stress distribution (Additional file 4: cases 5 and 6). At the growth plate of the balanced pelvis (case 6), the shear and compression stresses were respectively 17.4% and 5.1% higher than the retroverted pelvis (case 5). In the intervertebral disc, the shear stress was quite similar between the two cases. The stress increase between the balanced and retroverted pelvis was in the order of 8.1%.

Significant correlations were found between pelvic incidence and the resulting stresses (shear and compression stress in the growth plate and shear stress in the intervertebral disc) with p-value< 0.05 (Additional file 5). In addition, a significant correlation was found between the slip percentage and the shear stress found in the intervertebral disc. In contrast, no correlation was found between compression and shear stresses in relation to the sacral slope. Similarly, there was no relation between stresses in the growth plate and the induced slip percentage (p>0.05).

## Discussion

In the present study, a shift in measured stresses in the lumbosacral junction was observed. These results are in accordance with clinical observations made by Roussouly et al. who made the association between shear stress and slippage at the L5-S1 disc [18]. In the model, the shear and normal stress distribution on the growth plate and intervertebral disc became greater at the anterior portion of the lumbosacral junction as the grade of spondylolisthesis increased. Stresses in simulated low grade cases occurred in front of the sacral dome and on the posterior part of the growth plate. The stresses moved to the anterior part of the intervertebral disc and growth plate for the simulated higher grade configurations. The increase of the compression stress could be explained by the more gravitational (vertical) loads sustained by the case with the smallest SS. The increased shearing stress might be coming from the need of global posture balance. From statics' point of view, the patient's body topology, body weight, and the presence of obesity are also important factors of the intervertebral stresses. Further studies will be needed to fully understand this particular scenario.

The lumbosacral junction is an important area in the study of the slippage of L5. The spondylolisthesis causes a sagittal unbalance, so that forces (gravity and weight) are concentrated more on the anterior part of the lumbosacral junction and thus create more stresses and load in this area [13]. Therefore, differential stresses on the anterior vs. middle and posterior part of its growth plate could modify the bone growth rate distribution according to the Hueter-Volkman principle and may lead to a doming of the sacrum [34-38]. This sacral dome could then further increase sliding of the L5 vertebra and progression of the spondylolisthesis.

In addition, we have documented that the increase of stresses was related to the increase of slip percentage, supporting previous reports in the literature [39,40]. Although no validated physiological stress limits for lumbosacral intervertebral elements exist in the literature, and the limits should be, from the authors' opinion, highly individual-dependent, it is probable that the anterior stresses reported in this study exceed the maximum value of physiological stresses reported in the literature [41,42]. Based on the compression stress at the posterior part of the sacral endplate, we can anticipate that it could promote the formation of a sacral dome as discussed in the previous paragraph. The unbalance of forces promotes an increase of bone growth on the sacrum and as a result the formation of the sacral dome. This formation explains the stress concentration on the dome for the compression. This also explains why the increase of stresses at the lumbosacral junction could lead to the formation of a sacral dome and therefore to further changes in pelvic incidence and sacral slope [7,13,17]. In the framework of the Hueter Volkmann principle, the increase of compression at the anterior and posterior border of S1 endplate could promotes the growth of the osseous doming and aids the sliding of L5 vertebra, and consequently the risk of progression [34-38,43]. Moreover, shear stress at the L5-S1 disc could further promote the slippage. The increased shear stress at the anterior surrounding area of the lumbosacral junction was 10% higher than the compression stress, so, their combined effect favors the sliding of the vertebrae and strengthens the idea that these types of stresses are associated with the risk of progression as hypothesized in previous reports [8,18,39].

This study had also demonstrated that there are significant correlations occurring between stresses and pelvic incidence, as well as with slippage of the vertebra. Since the spino-pelvic interaction is closely related to the stresses at the spino-pelvic junction, the correlation between the stresses and the spino-pelvic parameters revealed in this study can be used for predicting spondylolisthesis progression [44-49]. This study, along with others, suggests that pelvic incidence is an important biomechanical parameter to predict progression of spondylolisthesis [1,2,50].

The model used in this study has several limitations which need to be recognized when interpreting the results. For instance, the materials properties of spinal tissues were not specific to spondylolisthesis cases but taken from published values from cadaver spines [16,22,25-27,51]. Differences in the disc and bone stiffness may affect the stress distribution [18]. The model allows only the study of the immediate distribution of stress at the lumbosacral junction in a given posture and not the dynamic and long term response which occurs under growth, change of posture, physical daily activities, etc. The complete validation of such a model, as any FEM, is difficult. The partial assessment using the published results of Sairyo however provides confidence in the results and interpretation presented herein [8,9]. The study was based on one real spondylolisthesis case. The 10 patterns with various % slip and spino-pelvic were generated from this initial geometry (parametric model). The normal geometry of this patient was therefore unavailable for this study, as well as the normal L5 pars interarticularis of this patient was not documented by any medical imaging. Some of the findings from this study were specific to the particular case used, putting additional limitations to this study. Simulations of an additional number of cases are necessary to generalize the findings. Since the biomechanical properties of the intervertebral elements undergoing degeneration are likely to be dependent on the degree of degeneration as a function of 3D location, the use of high field strength MRI in conjunction with biomechanical testing is envisaged to improve the model precision. The results of future biomechanical analysis could be compared to and presented side-by-side with clinical observations to provide clinicians with the insight of the related biomechanics in a more informative way.

## Conclusions

The developed modeling approach is the only tool at the moment which enables to interpret biomechanically the spino-pelvic parameters in connection with the biomechanics of spondylolisthesis. The parameterization of different spino-pelvic parameters in a FEM is a first step in the modeling of spondylolisthesis. It allows analyzing and understanding the biomechanics according to different configurations of the pathology. Future biomechanical analyses of patient-specific cases, in addition to clinical assessment using radiographs, could offer complementary perspectives on the understanding of stress distribution that could lead to further progression of the deformity. This combined approach could eventually help surgeons to predict the spondylolisthesis progression in the clinical context and therefore to better plan and prepare the surgical treatment.

## List of abbreviations

PI: Pelvic incidence; SS: sacral slope; PT: pelvic tilt; FEM: finite element model; BW: body weight

## Competing interests

The authors declare that there are no financial and non-financial competing interests related to the publication of this manuscript.

## Authors' contributions

AS, CA, and HG have made substantial contributions to the study design, computer modeling, numerical simulations, analysis and interpretation of data. XW and HL have significantly contributed in drafting the manuscript and revising it critically for important intellectual content. All authors read and approved the final manuscript

## Supplementary Material

Additional file 1 **table_1_sevrain_v_1.doc**. Click here for file

Additional file 2 **table_1_sevrain_v_2.doc**. Click here for file

Additional file 3 **table_1_sevrain_v_3.doc**. Click here for file

Additional file 4 **table_1_sevrain_v_4.doc**. Click here for file

Additional file 5 **table_1_sevrain_v_5.doc**. Click here for file
