# Supplementary material for: Biomechanical evaluation of predictive parameters of progression in adolescent isthmic spondylolisthesis: a computer modeling and simulation study
Source: Scoliosis. 2012 Jan 18;7:2. doi: 10.1186/1748-7161-7-2 (PMC3283472; doi:10.1186/1748-7161-7-2)
Supplement: Additional file 1 — table_1_sevrain_v_1.doc. [file 1748-7161-7-2-S1.DOC]

| Segments | Tissues | | Stiffness modulus (N/mm) | Elastic modulus (MPa) | Poisson coefficient | References |
| --- | --- | --- | --- | --- | --- | --- |
|  |  |  | k | E_x_ | µ |  |
| T1-L3 | Vertebral body | | - | 1000 | 0.3 | Aubin et al. (1995) Beauséjour et al. (1999) Huynh et al. (2007) |
|  | Pedicle | | - | 5000 | 0.3 |  |
|  | Spinous process | | - | 3500 | 0.3 |  |
|  | Lamina | | - | 1000 | 0.45 |  |
|  | Ribs | | - | 5000 | 0.45 |  |
|  | Cartilage | | - | 480 | 0.45 |  |
|  | Sternum | | - | 10000 | 0.45 |  |
|  | Intervertebral disc | | - | 3.12-17.75 | 0.45 |  |
|  | Articular facets | | 500 | - | - |  |
| L4-Pelvis | Cortical bone | | - | 8000 (E_y_=8000, E_z_=14000, G_x_=G_y_=G_z_=3280) | 0.3 | El-Rich et al. (2008) |
|  | Trabecular bone | | - | 767 (E_y_=40, E_z_=1157, G_x_=20,4, G_y_=G_z_=24) | 0.12 |  |
|  | Ligaments – area (mm²) | |  | | |  |
|  |  | Anterior Longitudinal – 38 | - | 20 | 0.3 |  |
|  |  | Posterior Longitudinal - 20 | - | 70 | 0.3 |  |
|  |  | Intertransverse – 10 | - | 50 | 0.3 |  |
|  |  | Flavum – 60 | - | 50 | 0.3 |  |
|  |  | Capsular – 40 | - | 20 | 0.3 |  |
|  | Intervertebral disc | |  | | |  |
|  |  | Nucleus Pulposus | - | 2 | 0.499 |  |
|  |  | Annulus ground | - | 8 | 0.45 |  |
|  |  | Annulus fiber | 904 | - | 0.3 | Iatridis & Gwynn (2004) |
|  | Growth Plate | | 10 | - | 0.4 | Konz et al. (2001) |
|  | Articular facets | | 500 | - | - | Aubin et al. (1995) |

Table 1: Material properties of the finite element model
