# Supplementary material for: Biomechanical evaluation of predictive parameters of progression in adolescent isthmic spondylolisthesis: a computer modeling and simulation study
Source: Scoliosis. 2012 Jan 18;7:2. doi: 10.1186/1748-7161-7-2 (PMC3283472; doi:10.1186/1748-7161-7-2)
Supplement: Additional file 2 — table_1_sevrain_v_2.doc. [file 1748-7161-7-2-S2.DOC]

| Vertebra | Body weight distribution (%) | Vertebra | Body weight distribution (%) |
| --- | --- | --- | --- |
| T1 | 14 | T10 | 38.7 |
| T2 | 16.6 | T11 | 42 |
| T3 | 19.2 | T12 | 44.8 |
| T4 | 21.8 | L1 | 47.6 |
| T5 | 22.7 | L2 | 50.4 |
| T6 | 26 | L3 | 53.2 |
| T7 | 29.3 | L4 | 55.5 |
| T8 | 32.6 | L5 | 57.8 |
| T9 | 35.9 |  |  |

Table 2: Body weight distribution on each vertebral body
