# Supplementary material for: Biomechanical evaluation of predictive parameters of progression in adolescent isthmic spondylolisthesis: a computer modeling and simulation study
Source: Scoliosis. 2012 Jan 18;7:2. doi: 10.1186/1748-7161-7-2 (PMC3283472; doi:10.1186/1748-7161-7-2)
Supplement: Additional file 3 — table_1_sevrain_v_3.doc. [file 1748-7161-7-2-S3.DOC]

|  | inferior modality | Intermediate modality | superior modality |
| --- | --- | --- | --- |
| Pelvic Incidence | 52° | 60° | 75° |
| Sacral Slope | 45° | -- | 60° |
| % Slip percentage | 40% | 60% | 80% |

Table 3: Modalities of the spino-pelvic parameters for the design of experiments
