# Supplementary material for: Biomechanical evaluation of predictive parameters of progression in adolescent isthmic spondylolisthesis: a computer modeling and simulation study
Source: Scoliosis. 2012 Jan 18;7:2. doi: 10.1186/1748-7161-7-2 (PMC3283472; doi:10.1186/1748-7161-7-2)
Supplement: Additional file 4 — table_1_sevrain_v_4.doc. [file 1748-7161-7-2-S4.DOC]

| Test no. | PI | SS | slip % | τ_maxGP_ | τ_maxD_ | σ_maxGP_ |
| --- | --- | --- | --- | --- | --- | --- |
| 1 | 52 | 45 | 40 | 7.2 | 5.2 | -15.6 |
| 2 | 75 | 45 | 40 | 8.3 | 5.5 | -17.4 |
| 3 | 52 | 60 | 40 | 7.3 | 5.3 | -15.8 |
| 4 | 75 | 60 | 40 | 8.8 | 5.5 | -18.1 |
| 5 | 75 | 45 | 80 | 12.3 | 5.4 | -23.3 |
| 6 | 75 | 60 | 80 | 14.4 | 5.6 | -24.5 |
| 7 | 52 | 45 | 60 | 10.5 | 5.7 | -21.8 |
| 8 | 60 | 45 | 80 | 13.9 | 6.2 | -27.5 |
| 9 | 52 | 60 | 60 | 9.4 | 5.8 | -19 |
| 10 | 60 | 60 | 80 | 7.9 | 2.2 | -15.2 |

Table 4: Stresses (MPa) resulting from the ten simulated configurations (τ_maxGP_: Maximal shear stress for the growth plate of S1; τ_maxD_: Maximal shear stress of the intervertebral disc of L5-S1; σ_maxGP_: Maximal compression stress of the growth plate of S1, PI: Pelvic Incidence, SS: Sacral Slope, %: Slip percentage)
