# Supplementary material for: Biomechanical evaluation of predictive parameters of progression in adolescent isthmic spondylolisthesis: a computer modeling and simulation study
Source: Scoliosis. 2012 Jan 18;7:2. doi: 10.1186/1748-7161-7-2 (PMC3283472; doi:10.1186/1748-7161-7-2)
Supplement: Additional file 5 — table_1_sevrain_v_5.doc. [file 1748-7161-7-2-S5.DOC]

|  | PI | SS | Slip % |
| --- | --- | --- | --- |
| τ_maxGP_ | 0.05 | 0.86 | 0.18 |
| τ_maxD_ | 0.01 | 0.09 | 0.05 |
| σ_maxGP_ | 0.04 | 0.51 | 0.23 |

Table 5: ANOVA results (p- values) describing the effects of the spino-pelvic parameters on the maximal shear stress at the growth plate of S1 and the intervertebral disc of L5-S1, as well as the Maximal compression stress of the growth plate of S1
